# Dental anxiety and dental care - a comparison between Albania and Germany

**DOI:** 10.1186/s12903-024-04887-2

**Published:** 2024-09-23

**Authors:** Nertsa Cunoti, Rezart Qorri, Lisa Irmscher, Erda Qorri, Laura Magerfleisch, Hendrik Berth

**Affiliations:** 1 Division of Psychological and Social Medicine and Developmental Neurosciences, Research Group Applied Medical Psychology and Medical Sociology, Faculty of Medicine, TUD Dresden University of Technology, Fetscherstraße 74, 01307 Dresden, Germany; 2 Department of Dentistry, Faculty of Medical Sciences, Albanian University, Tirana, 1001 Albania

**Keywords:** Anxiety, Dental anxiety, Dental clinic, Depression, Somatization, Psychological distress, Albania, Germany, Oral health, Dental care

## Abstract

**Background:**

The present study is the first in Albania on dental fear and dental anxiety and also in the field of psychosocial medicine. The purpose of this study was to find out whether there are differences in dental anxiety using the Dental Anxiety Scale, their level of psychological distress using the Brief Symptom Inventory-18 and the evaluation of oral health among Albanian and German patients.

**Methods:**

This study was conducted in the period from December 2019 to July 2020, a sample of *N* = 263 patients (133 Germans, 130 Albanians) using the Dental Anxiety Scale questionnaires to determine anxiety before dental treatment and the Brief Symptom Inventory-18 to evaluate psychological distress. Moreover, the patients answered questions regarding their oral health and dental care. In Germany, there were four refusals to entrance in the study due to various reasons, in contrast to Albania, where there were no refusals at all For the purposes of this study, data on both populations aged 14 years and older were used.

**Results:**

The questionnaires results were calculated for all participants. The current subjective health status of Albanian patients was assessed to be significantly worse than that of German patients (*p* < 0,000). Germans were more susceptible to signs of Anxiety (*p* < 0,000), Depression and Somatization and scored higher on the Dental Anxiety Scale and the Global Severity Index (*p* < 0,000) than Albanian patients. Additionally Albanian patients scored significantly lower on the preventive care index (*p* < 0,000). Despite an elevated DAS anxiety level, German patients reported going to the dentist more frequently than Albanian patients.

**Conclusion:**

The results showed that between both populations differences in dental anxiety, psychological distress and oral health exists. Patients from Germany report more psychological distress and described more dental anxiety compared to Albanian patients. Albanian patients reported not utilization on oral health care.The implementation of educational programs and preventive measures, would contribute to raising awareness about the importance of oral health and increased use of dental services.

## Background

Despite constant medical advances, dental anxiety remains a condition that is reversible through desensitization of the general population. Nearly 80% of adults in industrialized countries experience discomfort before dental treatment, with 20% expressing a genuine fear of dental procedures, and 5% actively avoiding dental care altogether [1]. The prevalence of dental anxiety is evident across all age groups, with even young children exhibiting avoidance behaviour towards dental treatment, often influenced by parental attitudes [2].

An extreme manifestation of dental anxiety is termed dental phobia, which can be classified using the International Classification of Diseases (ICD). According to the ICD-10 Chapter V, F40.0, a phobia falls under the category of anxiety disorders. It is characterized as an irrational fear of a specific, generally non-threatening situation that is either completely avoided or endured with significant distress [3]. This classification is distinct from phobias related to specific stimuli encountered during dental treatment, such as injections [4]. One way to differentiate between anxiety and phobia stages is by assessing their impact on the individual’s daily routine and life. If it disrupts social life, occupation, and normal functioning, it may be considered a specific (dental) phobia [5]. Dental anxiety proves stressful for both the patient and the dentist, leading to reduced cooperation, prolonged treatment times, and an uncomfortable treatment environment [6]. This can lead to inaccurate diagnosis and inappropriate treatment, including the evaluation of tooth vitality [7]. Patients who entirely avoid dental care often suffer from poor dental and periodontal health [8]. Such individuals typically seek dental attention only when the pain becomes unbearable, requiring complex interventions like root canal therapy or extractions. This perpetuates a negative cycle that undermines the development of a healthy dentist-patient relationship [9]. Factors such as the dental clinic environment, stress experienced during procedures, cognitive capacities of the individual, and cultural practices are known to influence dental fear and anxiety (DFA) [10]. DFA poses daily challenges for dentists treating both children and adults. In pediatric dentistry, with a prevalence of 9%, DFA significantly complicates patient management [11, 12]. In adults, dental fear and anxiety often reflects past negative dental experiences from childhood or adolescence. Dental fear is an acute, distressing response to perceived threats [13, 14].

Studies from various countries have reported dental fear and anxiety prevalence rates of 12.5% in Canada [15], 12.6% in Russia [16], 13.5% in France [17], 16.1% in Australia [18], and 30% in China [16]. Research in Saudi Arabia indicates DFA rates among adults range from 27 to 51% [10, 19], among children, the rates range from 43,1% to 47,6% [20, 21]. DFA can hinder the use of dental services, impacting early disease detection and management. Among children in Eastern Europe, significant levels of anxiety were reported, with varying rates across countries. A total of 12.5% of children from Croatia, 26.67% from Macedonia, 10.94% from Bosnia and Herzegovina, 20.31% from Montenegro, 23.08% from Slovenia and 16.10% from Serbia showed a high level of anxiety [22].

An observational study in Albania involving 180 participants aged 15 to 55 found that 70% displayed high dental fear regarding orthodontic treatments and fillings, 59% towards dental implants, and 74% exhibited extreme fear of extractions [23]. 64% of the surveyed participants reported having gingivitis, and 61% indicated they suffered from dental caries, in contrast to 53% who had undergone tooth extractions. The data analysis revealed that tooth extractions and dental caries significantly affected high blood pressure, with a P ˂ 0.0001 [23]. Taheri et al. explored the connections between dental pain perception and its relationship to pain anxiety, dental anxiety, and mental pain, finding significant correlations (*p* = .001) between pain perception with dental anxiety (*r* = .38), pain anxiety (*r* = .45), and mental pain (*r* = .25) [24].

The approach to a dentist’s office significantly influences dental fear scores. Patients often view surgical and restorative procedures as unpleasant and intimidating, with past negative experiences potentially exacerbating fears during subsequent visits [25]. Furthermore, previous negative experiences in the dental office can instigate fear during subsequent visits. Dental anxiety (DA) is more intense and irrational compared to general fear [22]. This type of anxiety leads patients to avoid treatment, reflecting a shortfall in modern dentistry’s evolution toward minimal invasiveness. Although modern anesthetics can minimize pain, the fear of pain often exceeds the actual sensation of pain. Anxiety disorders are widespread, with 25% of general practitioners recognizing symptoms in their patients [26].

Mental health is crucial, recognized by the WHO as a state where individuals achieve their potential, handle life’s normal stresses, work productively, and contribute to their community [27]. Recently, mental disorders and psychosocial disabilities have gained recognition as significant global development issues [28].

The WHO estimates that one in four individuals will encounter a mental health condition in their lifetime, with around 600 million people worldwide disabled due to mental health issues [29].

The public health significance of mental illnesses underscores their multifaceted causes, primarily rooted in social issues. In Albania, seeking and receiving psychological support often faces significant prejudice. The Albanian culture, characterized by extremes, readily accepts or rejects things. In this regard, there is a notable lack of empathy, emphasizing the need for an improved attitude among Albanians toward psychological services and mental health. As the stigma surrounding mental health continues to decrease, more individuals are seeking professional help for their mental health issues. This trend is driving the growth of therapy and counseling services in the country [30].

Comparing the Albanian healthcare system to the German counterpart, dental treatment is guaranteed for specific citizens only through public dental health services, excluding all private clinics [31].

The Ministry of Health has approved free fluoridation for all children up to the age of 18, although this service is underutilized. In Albania, health insurance covers only dental emergencies, primarily focusing on tooth extractions [31].

According to a survey conducted by the European Commission on the quality of life of Albanians, it was discovered that 41% of Albanians consistently postpone or entirely avoid visiting the doctor in order to save money [32]. Consequently, dental care is not easily accessible, as evidenced by half of those surveyed in Albania stating that they either never visit the dentist or only seek dental care when the pain becomes unbearable [32]. A study in Kosovo conducted that in total, 2,556 school children the caries prevalence for 7- to 14-year- old school children was 94.4% [33].

The healthcare system in Germany offers a variety of options for dental care. As with all medical services, the statutory health insurance covers the cost of treatments only if the patient consults a dentist who is accredited to provide contracted dental care [34].

Dental services in Germany include: (1) An annual check-up, (2) Dental care for children and adolescents from six months to 17 years old, (3) Oral health services for individuals with disabilities or those in need of nursing care, (4) General dental treatments primarily include the removal of tartar, fillings, root canal treatments, oral surgery, periodontal services, and treatments for oral mucosal diseases. These services are generally free of co-payments for the insured, (5) Orthodontic treatments until the age of 18, (6) Costs for dental prostheses [34].

In 2021, the German Dental Association (BZÄK) outlined the oral health goals for Germany’s health system for 2030, based on robust epidemiological evidence [35]. The 2030 agenda includes both disease-oriented and health-promotion goals. Key targets are achieving a caries-free rate of 90% among 3-year-olds and 12-year-olds, reducing the prevalence of severe periodontal disease to below 10% in middle-aged adults (35–44 years old), and enhancing oral health-related behaviors [35]. Behavioral objectives aim to increase the frequency of twice-daily toothbrushing to 87.5% among children, 85.3% among adults, and 89.1% among seniors. Additionally, the agenda seeks to increase the proportion of individuals who attend regular dental check-ups annually to 86.9% for children, 75% for adults, and 94.6% for seniors [35, 36].

This marks the first Albanian scientific study in the fields of dentistry and psychosocial medicine and no prior research had explored dental anxiety. Consequently, we initiated a study to address this gap in the existing literature. The objective of this study is to investigate potential differences in dental anxiety between individuals from Albania, categorized as a “third country” and Germany, classified as an “industrialized country”. Additionally, the study aims to compare the dental care systems of both countries. Special emphasis is placed on assessing the anxiety levels of dental patients during a single visit to a clinic in Germany and Albania, with the overarching goal of identifying and comparing preventive behaviors and oral health status among these groups.

## Methods

In Plauen, Germany and in Tirana, Albania the research group consists of dentists from both countries, collected data over the course of eight months (12.2019–07.2020). The questionnaires included various instruments such as the Dental Anxiety Scale (DAS) [37], the Brief Symptom Inventory-18 [38], and a set of descriptive questions gathering information about preventive behavior and oral health status, were handed out to a total of *N* = 263 patients, 133 patients from a private dental clinic in Plauen, Saxony (Germany), and 130 patients from the dentistry university clinic in Tirana (Albania) before treatment. The age range of participants varied from 14 to 80 years. All patients had to voluntarily take part in this study. The study was divided into two groups: Albanian and German patients. They were selected based on their explicit admission, made at the reception, of being afraid of the dentist. They were required to complete our questionnaires before treatment in the dental clinic in the waiting room. The questionnaire was administered by the dentists, who distributed it to the patients. All questions are designed to be easily understandable and free of medical jargon to avoid misunderstandings.

The method of questioning was consistent and systematically applied to all participants. A structured and constant procedure ensures that all respondents are treated the same way, which is crucial for the validity and reliability of the results. In Germany, there were four refusals to participate in the study due to various reasons, in contrast to Albania, where there were no dropouts. The questionnaires were then examined in 2020 by our research group.

Other inclusion criteria included having sufficient knowledge of the German and Albanian languages, possessing the physical and mental ability to complete the questionnaires, being oriented in terms of time and place, and displaying no psychiatric symptoms. This study did not involve a specific screening for psychological problems, and dental phobia or a high level of dental anxiety were not considered exclusion criteria. All patients provided written informed consent, and only those patients who gave written informed consent were included as study participants. For individuals younger than the age of 18 consent to participate were obtained from their parents or legal guardians. For Albanian patients, the German (validated) versions of the scales were utilized, and they were translated into the Albanian language by translators by hand when necessary.

### Statistical procedure

All questionnaires underwent analysis using the statistical program ‘Statistical Package for the Social Sciences’ (SPSS). Mean total values were calculated and subsequently analyzed using an independent sample t-test. Chi-squared tests were employed to ascertain significance between questionnaire categories and sample characteristics. The level of significance was set at *p* < .05. The required sample size was determined using G*Power 3.1.3 [39]. For comparing two groups with T-Tests (two independent means, two-tailed), with a significance level of α = 0.05, an effect size of Cohen’s d = 0.5, and a power of 95% (1- β = 0.95), a sample size of at least *N* = 105 per group (total *N* = 210) was necessary.

### Dental anxiety scale

The Dental Anxiety Scale (DAS) was initially introduced in 1969 by Corah and is widely utilized for assessing dental fear in patients [37, 40]. The total dental anxiety score is calculated by summing up the scores from the four questions. The scores range from 4 to 20, and the patient’s level of anxiety is quantified as follows: a total score of 4 indicates “no fear”, a score between 5 and 8 corresponds to “low fear”, a score between 9 and 14 indicates “moderate fear”, and a score between 15 and 20 corresponds to “high fear” [41]. These scores help evaluate the level of dental anxiety experienced by the patient. The reliability of the Dental Anxiety Scale was found to be rtt = 0.86 [37]. In this study, Cronbach’s alpha was calculated as 0.76 (N = 263). The questionnaire was chosen to assess dental anxiety in this study due to its brevity and scientifically proven reliability.

### Brief symptom Inventory-18

The Brief Symptom Inventory-18 (BSI-18) was introduced in 2000 by Derogatis [42] as a further condensed version of the BSI, which originally comprised 53 items from the Symptom-Checklist 90-R. Developed to assess the state of psychological stress with only 18 items [43], the BSI-18 has been applied in various contexts, including with cancer patients, victims of terrorist attacks, individuals with posttraumatic stress, those dealing with alcohol addiction, and other populations. The three scales—depression, anxiety, and somatization—each consist of six items and contribute to the Global Severity Index (GSI). Scores can range from 0 to 90, with each of the 18 items reflecting the respondent’s experiences over the last seven days on a scale offering four choices from ‘Not at all’ to ‘Extremely.’ The reliability of the three scales was assessed in 2010 on a sample of 638 psychotherapeutic patients: somatization α = 0.79, depression α = 0.84, anxiety α = 0.84, and GSI α = 0.91 [44]. In our study, the reliability of the different BSI-18 scales was as follows: somatization α = 0.78, depression α = 0.72, anxiety α = 0.81, and GSI α = 0.90.

### Oral health

In this study, patients were asked to provide answers to questions regarding their assessment of oral health and dental care. Questions followed:

1. How many times a day do you brush your teeth? (Never, 1x/day, ≥2x/day)
2. How often do you go to the dentist? (For example, for prophylaxis). (Never, 1x/year, ≥2x/year)
3. How often do you have tartar removed? (Never, 1x/year, ≥2x/day)
4. How often do you have a professional teeth cleaning appointment? (Never, 1x/year, ≥2x/day)
5. How much do you think you can do to maintain the health of your teeth? (Nothing at all, little, some, much, very much)

## Results

The mean score for the patients’ current subjective overall health was 2.49 (SD 1.18). The patients’ Dental Anxiety Scale (DAS) averaged 13.10 (SD 2.74). Consequently, the psychological distress of the patients, as assessed by the BSI-18, revealed mean values of 3.45 (SD 3.95) for the anxiety scale, 2.10 (SD 3.00) for the depression scale, and 2.56 (SD 3.31) for the somatization scale. The global trait score GSI had a mean of 8.11 (SD 9.13).

**Table 1:** Mental health status - a comparison between Albania and Germany

	Total Group (*n* = 263) M (SD)	Albania (*n* = 130)	Germany (*n* = 133)	t-Test
**Overall health**	2,49 (1,18)	2,78 (1,42)	2,20 (0,77)	t (df = 198,36) = -4,166, *p* < 0,**000** d = − 0.517
**Dental Anxiety (DAS)**	13,10 (2,74)	12,62 (2,44)	13,58 (2,94)	t (df = 261) = 2,890, *p* = 0,**004** d = 0,356
**Anxiety (BSI-18)**	3,45 (3,95)	1,83 (2,52)	5,03 (4,43)	t (df = 210,39) = 7,210, *p* < 0,**000** d = 0,884
**Depression (BSI-18)**	2,10 (3,00)	1,65 (2,41)	2,54 (3,42)	t (df = 261) = 2,446, *p* = 0,**015** d = 0,302
**Somatization (BSI-18)**	2,56 (3,31)	1,99 (2,54)	3,12 (3,85)	t (df = 228,86) = 2,811, *p* = 0,**005** d = 0,345
**Overall psychological distress (GSI**,** BSI-18)**	8,11 (9,13)	5,47 (6,56)	10,69 (10,48)	T- (df = 222,45) = 4,857, *p* < 0,**000** d = 0,596

Table 1 presents a comparison of patient groups interviewed in Albania and Germany concerning their psychological well-being. The t-test results indicate a significant difference between the two patient populations across all measures, with effect sizes (Cohen’s d) falling within the medium to high range. Statistical analysis revealed that Albanian patients rated their overall health worse than German patients. Additionally, significant differences emerged between the two groups in responses to the Dental Anxiety Scale (DAS), with Germans reporting higher levels of dental anxiety. Furthermore, it became evident that German patients experience significantly more psychological distress, as observed across the depression, somatization, and anxiety subscales.

**Table 2:** Screening behavior and oral health status - a comparison between Albania and Germany

		Albania (*n* = 130) *n* (%)	Germany (*n* = 133) *n* (%)	Chi-square test/ t-test
**How often do you brush your teeth?**	never 1x/day ≥ 2x/day	11 (8,5) 46 (35,4) 73 (56,1)	2 (1,5) 24 (18,0) 107 (80,5)	Chi-Square (df = 4) = 24,775, *p* < 0,**000**
**How often do you go to the dentist?**	never 1x/year ≥ 2x/year	7 (5,4) 65 (50,0) 58 (44,6)	14 (10,5) 43 (32,3) 76 (57,2)	Chi-Square (df = 4) = 14,549, *p* = 0,**012**
**How often do you have tartar removed?**	never 1x/year ≥ 2x/year	39 (30,0) 68 (52,3) 23 (17,7)	29 (21,8) 67 (50,4) 37 (27,8)	Chi-Square (df = 4) = 6,898, *p* = 0,141
**How often do you have a professional teeth cleaning appointment?**	never 1x/year ≥ 2x/year	72 (55,4) 42 (32,3) 16 (12,3)	57 (42,9) 50 (37,6) 26 (19,5)	Chi-Square (df = 4) = 7,773, *p* = 0,169
**How much can you do yourself to maintain or improve the health of your teeth?**	nothing at all little some a lot very much	0 (0,0) 20 (15,4) 57 (43,8) 49 (37,7) 4 (3,1)	1 (0,8) 4 (3,1) 25 (19,1) 56 (42,1) 47 (34,9)	Chi-Square (df = 4) = 58,924, *p* < 0,**000**

Table 2 provides a comparison of the oral health status and preventive behaviour between the two patient groups. Patients in the Albanian group reported brushing their teeth significantly less often than their German counterparts. Correspondingly, German patients also visited the dentist significantly more frequently than Albanians. In terms of tartar removal and professional teeth cleaning, there is a descriptive difference between German and Albanian participants, with Germans undergoing these treatments more frequently, although this result did not reach statistical significance.

Additionally, a significant difference was observed in the perceptions of the two groups regarding their contribution to the health and maintenance of their own teeth. The majority of German subjects (75.9%) believed they could contribute a lot or very much to their own oral health, whereas in the Albanian group, only 40.8% thought the same, indicating a significant difference.

## Discussion

This is the first study to investigate the prevalence of dental anxiety and mental health problems in Albania and compare it with Germany. Due to the sample size and the study’s restricted scope to one city in Germany (Plauen/Saxony) and the capital of Albania (Tirana), it’s important to note that the data, including the study results, may not be fully representative of the entire populations of Germany and Albania.

In this study the mean value for the Dental Anxiety Scale (DAS) in the patient collective was 13.10 (2.74). However, when comparing the expression of dental treatment anxiety, significant differences between the patient groups specifically, the German and Albanian subjects were observed. The average DAS value was higher for Germans and slightly lower for Albanian patients. Notably, the DAS values of the German group exceeded the German average value established by Kunzelmann and Dünninger (1990) [45]. Thus, the study participants, in terms of the expression of their dental treatment anxiety, fall within the German average, with a significant value. The German findings align with those of other industrialized nations: in France, an estimated 13.5% of people suffer from moderate to severe dental anxiety [46], in Europe [47], in North America [48], and in Australia [49] (10–18%) but significantly lower than in countries like China, where the rate is 30% [50].

This study emphasizes the need for preventive measures against dental anxiety. Since dental anxiety often begins in childhood, young patients should be the primary focus of prevention efforts [51–53]. Early education has been shown to positively impact dental anxiety, leading to better long-term dental care [54]. Despite the strong correlation between dental anxiety and general state anxiety [55], patients frequently describe dental anxiety as an iatrogenic outcome of dental treatment [56]. This highlights the responsibility of the dental profession and individual practitioners.

Additionally, this study could advocate for the establishment of access centers for individuals with dental fear, particularly in Albania. Addressing dental fear requires a multidisciplinary team and is time-intensive. Training and rehabilitation are feasible in a supportive environment [57, 58]. In Northern Europe [59, 60], specialized units with multidisciplinary skills and defined protocols provide prevention and treatment for anxious patients. However, Albania lacks such teams, although there are developments in behavior management and sedation techniques. Furthermore, dental anxiety is often viewed as an inevitability rather than a treatable condition, despite classifications based on DSM-IV psychiatric criteria [61]. Consequently, there is little motivation to develop specialized services. For patients who do access the limited centers addressing both dental fear and dental disease, the costs are not covered by social security, exacerbating oral health inequalities for those with dental anxiety in Albania.

Nevertheless, this study revealed a generally higher level of psychological distress using the Brief Symptom Inventory-18 (BSI-18). In terms of psychological distress, significant differences were observed between the two groups on all subscales as well as the Global Severity Index (GSI), with Germans reporting higher levels of psychological distress. In a sample of patients with anxiety disorders most comparable to ours, the following values for Cronbach’s alpha were found for the BSI subscales: somatization = 0.79, depressiveness = 0.87, and anxiety = 0.81 [62]. In our study, the corresponding values were 0.78, 0.72, and 0.81, respectively, suggesting that the BSI-18 is nearly identical.

However, the average score for both patient groups on the “Somatization” subscale was higher than the average values reported by Spitzer for a group of psychologically healthy individuals [63] On the “Anxiety” subscale, only the average score of the German patients was higher than these values, while the score on the “Depression” subscale for Albanian patients was lower than that of German patients [63]. The reasons for these findings in the German population are detailed in the following sections: Data from the 2015 Health Monitoring of the Robert Koch Institute (RKI) shows that in Germany at that time, nearly one in four men (22.0%) and nearly one in three women (33.3%) between the ages of 18 and 79 had experienced fully developed mental disorders at some point. The most common mental disorders were anxiety disorders (15.3%) and depressive disorders (7.7%), followed by somatic disorders (3.5%) [64].

Despite the increasing demand for psychological services, there remains a stigma towards mental illness among Albanians. As a result, individuals often seek the assistance of a psychologist only when the problem has become very serious, and the issues, after having consulted various doctors, appear to be uncontrollable.

A comparison of the preventive care behavior of the two patient populations revealed that Albanian patients had a significantly lower preventive care score than German patients. This discrepancy is particularly evident in the frequency of tooth brushing and dentist visits, as well as in the frequency of tartar removal and professional dental cleaning.

The most common reason that school children visit the dentist was a toothache. A regular recall and check-up was rarely reported. Usually, were accompanied by their parents. Their first comments regarding their dental visit were “my child a terrible toothache all night” and “we couldn’t sleep at all.” The children with toothaches had bad experiences at the dentist and thus refused future visits. Even though there were dental offices in some of the schools in this study, they were often dysfunctional and poorly equipped. Often, there were no dentists specializing in the fields of pedodontics [33]. The present study in Kosovo showed also that the mean DMFT (5.8) of school children in Kosovo was higher in comparison with school children of the following developed countries: Netherlands (1.1), Finland (1.2), Denmark (1.3), USA (1.4), United Kingdom (1.4), Sweden (1.5), Norway (2.1), Ireland (2.1), Germany (2.6) and Croatia (2.6) (16). The mean DMFT of Kosovo’s children (age 12) was similar to the mean values in Latvia (7.7), Poland (5.1) and a group of 12- to 14-year-olds in Sarajevo, Bosnia, Albania(7.18) [33, 65, 66]. 

Surveys of schoolchildren and teachers revealed a lack of knowledge about oral health, making teachers ineffective as an educational tool on the subject [33].

Nevertheless, it’s essential to note that the two patient groups differed significantly from each other.Distinct differences between German and Albanian patients were identified, with 42.9% of German patients never having undergone professional teeth cleaning, compared to a higher figure of 55.4% for Albanian patients.

Similar to other dental treatments, professional teeth cleaning can evoke anxiety in certain patients as it involves the removal of impurities and tartar from the tooth surface. For many patients, the use of dental tools automatically triggers fear of associated pain. Another contributing factor could be that professional tooth cleaning is often considered a private service, not fully covered by statutory health insurance in Germany. The situation is even more challenging in Albania, where statutory health insurance funds do not contribute to oral health. In Albania, patients are required to bear the full cost of dental services themselves.

Meanwhile, an increasing number of statutory health insurance companies in Germany have acknowledged the significance of prophylactic services and offer support through subsidies, such as bonus programs. However, additional initiatives should be implemented, particularly in the realm of education and information dissemination about the importance of prophylactic treatments and dental cleanings. This is crucial for preventing periodontal diseases and arresting their progression, given that the development and progression of caries are strongly influenced by individual behavior [65].

Contrary to the hypothesis that individuals interviewed outside ‘developed’ countries might exhibit higher levels of anxiety and psychological distress due to potential avoidance of dental visits, this study did not confirm such a trend. The positive finding in the oral health-related survey, where the majority of both patient groups expressed confidence in their ability to maintain healthy teeth, is a significant step forward.

### Importance of the study

The study revealed that patients outside German dental practices did not exhibit increased anxiety levels. However, it underscores the continued relevance of dental anxiety in those settings. Given the potential for dental avoidance behavior leading to severe dental issues, there is a recommendation for heightened awareness of dental anxiety among Albanian dentists. It is advised that Albanian dentists familiarize themselves with their patients’ oral health, promptly identify and address dental phobias. Essential to this is comprehensive healthcare and risk assessment by both general practitioners and dentists to effectively inform and advise individuals about the risks associated with neglecting dental treatment and prophylaxis.

### Implications for the research

To conduct a more comprehensive investigation into dental treatment anxiety, additional studies should be undertaken with participants from non-European countries. It is also advisable to include the recording of DMF-T/S values and PSI for the involved patients. Given that dental anxiety frequently emerges in early childhood, conducting an extra survey focusing on dental anxiety among children and adolescents could be beneficial and pertinent for future research.

### Limitations

Individuals aged 18 and older autonomously completed all the questionnaires in this study, while those below the legal age were included solely with explicit parental consent obtained through signed declarations. This introduces the possibility that some patients may not have been entirely candid in their responses, potentially downplaying the seriousness of their answers to avoid being identified as having dental anxiety. It’s important to recognize that the dataset might not comprehensively reflect the prevalence of dental anxiety in the population, particularly as it could exclude severely phobic patients actively avoiding dental treatment. Furthermore, the questionnaires did not inquire about the type of treatment participants anticipated post-survey. Those in acute pain might already be psychologically vulnerable, expecting more discomfort, and consequently, exhibiting greater apprehension toward treatment compared to those anticipating routine dental check-ups.

## Conclusion

The study’s conclusion is that individuals interviewed in Albania tend to avoid visiting the dentist not due to anxiety or other psychological distress but because they underestimate the importance of oral health. In comparison, German patients exhibit higher levels of dental anxiety and other psychological distress, possibly because they visit the dentist more frequently and, consequently, have had more negative experiences. Nonetheless, both Albanian and German dentists should heighten their awareness of the topic of ‘dental anxiety’ to be better equipped in dealing appropriately with patients experiencing increased anxiety. Further studies are needed to reveal other factors related to dental anxiety and psychological distress. The findings of the present study call for early implementation of preventive dentistry elements, oral health knowledge especially in Albanian curricula.

## Data Availability

The datasets generated during and/or analysed during the current study are available from the corresponding author on reasonable request.

## Abbreviations

BSI-18: Brief Symptom Inventory-18
DAS: Dental Anxiety Scale
GSI: Global Severity Index, total Score of the Brief Symptom Inventory-18
DMFT: The oral health status Index .Decayed, Missing and Filled Teeth
